# Detection of human antibody responses to tick-borne *Neoehrlichia mikurensis*

**DOI:** 10.1007/s00430-026-00869-z

**Published:** 2026-03-12

**Authors:** Rima Alsalihi, Kerstin Andersson, Alaitz Aranburu, Christine Lingblom, Linda Wass, Catharina Lewerin, Marie Edvinsson, Andreas Mårtensson, Kenneth Nilsson, Elisabet Skoog, Anna-Carin Lundell, Beatrice Bergström, Anna Grankvist, Christine Wennerås

**Affiliations:** 1 Department of Infectious Diseases, The Sahlgrenska Academy, University of Gothenburg, Göteborg, Sweden; 2 Department of Clinical Microbiology, Sahlgrenska University Hospital, Göteborg, Sweden; 3 Department of Rheumatology and Inflammation Research, Institute of Medicine, The Sahlgrenska Academy, University of Gothenburg, Göteborg, Sweden; 4 Department of Hematology and Coagulation, Sahlgrenska University Hospital, Göteborg, Sweden; 5 Department of Internal Medicine and Clinical Nutrition, Institute of Medicine, The Sahlgrenska Academy, University of Gothenburg, Göteborg, Sweden; 6 Department of Medical Sciences, Section of Infectious Diseases, Uppsala University Hospital, Uppsala, Sweden; 7 Global Health and Migration Unit, Department of Women’s and Children’s Health, Uppsala University, Uppsala, Sweden; 8 Department of Medical Sciences, Sections of Clinical Microbiology and Infectious Diseases, Uppsala University, Uppsala, Sweden; 9 Department of Clinical Immunology and Transfusion Medicine, Sahlgrenska University Hospital, Göteborg, Sweden

**Keywords:** Antibody, Immunoglobulin G, Immunoglobulin M, *Neoehrlichia mikurensis*, Tick-borne disease

## Abstract

Tick-borne *Neoehrlichia mikurensis* is the cause of neoehrlichiosis, an infectious disease that features fever and vascular events. Compromised B-cell immunity is a risk factor for severe neoehrlichiosis, indicating the importance of antibodies in host defense. The development of serological assays has been hampered by the difficulty of culturing these intracellular bacteria. Here we present the first serological test for *N. mikurensis*, an ELISA for human IgM and IgG antibody responses to a P44/Msp2 protein of *N. mikurensis.* Serum or plasma from immunocompetent (*n* = 44) and immunosuppressed (*n* = 60) Swedish adults infected with *N. mikurensis*, with and without symptoms, were analyzed and compared with blood samples from non-infected immunocompetent individuals (*n* = 17). Sera from non-infected children (*n* = 23) and plasma from cord blood (*n* = 10) were also analyzed. Immunocompetent neoehrlichiosis adults had higher IgM and IgG antibody levels compared with immunosuppressed neoehrlichiosis patients treated with the B-cell suppressive agent rituximab. There were no significant differences in the IgM or IgG antibody levels between immunocompetent individuals with symptomatic versus asymptomatic *N. mikurensis* infection. Sera from healthy children contained high levels of IgM antibodies, despite no evidence of current infection with *N. mikurensis* nor previous exposure to tick bites as reflected by negative *Borrelia* serology. This IgM response was absent in cord blood plasma (*n* = 10), indicating it was not due to natural IgM antibodies. The high constitutional IgM antibody levels to *N. mikurensis* in children may explain why there have been no reports of pediatric neoehrlichiosis.

## Introduction

*Neoehrlichia* (*N.*) *mikurensis* is the cause of the emerging tick-borne disease neoehrlichiosis. The first cases of human infection with this tick-borne bacterial species were published in 2010 [1–3]. The classical neoehrlichiosis patient is middle-aged, has compromised B-cell immunity, and presents with fever, fatigue, localized pain and venous vascular events such as thrombophlebitis and deep vein thrombosis [4]. The bacteria have an affinity for vascular endothelial cells, which is the likely target of the infection in human beings [5, 6]. Immunocompetent patients infected with *N. mikurensis* can have a variegated clinical picture that may include fatigue, low-grade fever, chills, swollen ankles, stiff legs, neck pain, sudden deafness, tinnitus, and in severe cases, arterial vasculitis mimicking systemic rheumatic disease [5, 7]. There have also been several reports of asymptomatic carriage of the infection for extended periods of time [8–13]. Intriguingly, there are to date no known cases of *N. mikurensis* infection in children, which is unexpected since children are one of the major age groups afflicted by *Borrelia* infections [14]. Furthermore, both bacterial species are harbored and transmitted by *Ixodes ricinus* ticks, the main vector of human tick-borne disease in Europe.

*N. mikurensis* is an intracellular bacterium that does not grow outside of cells, which explains why routine culture-based microbiological methods are not applicable for diagnostic purposes [15]. At present, PCR of blood samples is the only method for diagnosing *N. mikurensis* infection and is available in a few reference laboratories in Europe. Although this infection was discovered in humans 15 years ago, there are still no serological tests described for *N. mikurensis*. A major challenge has been the uncultivatable status of the bacteria (outside of cell lines), which has made it difficult to obtain high-purity bacterial extracts for the development of antibody assays. It was only recently that the entire genome of *N. mikurensis* was sequenced and annotated [16], permitting in silico analyses of candidate bacterial proteins that could serve as antigens for serological tests. Antibody tests for *N. mikurensis* are sorely needed, not only as a useful diagnostic aid, but also to help answer many questions regarding the epidemiology and immunopathogenesis of this novel tick-borne infectious agent.

Here, we introduce the first serological test for human *N. mikurensis* infection, an ELISA for the detection of IgM and IgG antibodies against the outer membrane protein P44/Msp2 of *N. mikurensis*. This small protein of 19 kDa (174 amino acids) appears to be specific to *N. mikurensis*, as the closest homologue is harbored by the bacterial species “*Candidatus* Neoehrlichia lotoris” of raccoons and displays a mere 50% identity at the amino acid level. Importantly, P44/Msp2 proteins are an important target of the antibody response in humans infected with the related bacterial species *Anaplasma phagocytophilum* [17]. In this study, we present data on IgM and IgG antibody levels to P44/Msp2 of *N. mikurensis* in infected and non-infected Swedes of different age groups, with and without symptomatic *N. mikurensis* infection, and with and without compromised immunity.

## Materials and methods

### Human samples

Swedish biobanked blood samples from individuals enrolled in the following studies were analyzed: Plasma from immunocompetent and immunosuppressed patients with neoehrlichiosis (Dnr 394-12), plasma from patients with suspected vector borne-infections at the Center for Vector-borne Infections in Uppsala (Dnr 2015/249 and 2021/03390), plasma from Swedish blood donors participating in a prospective study of *N. mikurensis* infection (Dnr 2023-01604-01; 2025-01290-02), serum from control children enrolled in studies of allergy development (Dnr 298-12 and 366-14), and plasma from cord blood samples (Dnr 404-18). Table 1 summarizes the demographic data of the study subjects. The clinical pictures of the patients with neoehrlichiosis have been published previously [4–7, 18–20]. All studies had been approved by the Swedish Ethical Review Authority and were conducted in accordance with the ethical guidelines of the Declaration of Helsinki.

**Table 1:** Demographic characteristics of study subjects

Patients and samples	n	*N. mikurensis* infection	CT, median (25/75%)	Age, median years (Min–max)	Sex (% female)	References
*Immunocompetent individuals*						
Patients	22	Symptomatic	30 (28/33)	64 (28–83)	59	[5, 7]
Blood donors	22	Asymptomatic	30 (28/31)	54 (22–68)	23	–
Blood donors	17	Non-infected	–	53 (30–71)	53	–
Children	23	Non-infected	–	10 (2–17)	52	–
Cord blood	10	Non-infected	–	0	50	–
*Immunosuppressed individuals*						
Rituximab therapy^a^	45	Symptomatic	20 (18/29)	65 (32–87)	58	[4–7, 18–20]
Other therapies^b^	15	Symptomatic	29 (26/36)	66 (48–79)	53	[4–7, 18–20]

^a^For multiple sclerosis (31%); rheumatic diseases (22%); hematologic malignancies (20%); autoimmune conditions (20%), other diseases (7%)

^b^Azathioprine (n = 3), systemic corticosteroids (n = 3), gamma globulin substitution (n = 2), methotrexate (n = 1), venetoclax (n = 1), obinutuzumab (n = 1), belimumab (n = 1), mycophenolate mofetil (n = 1), ibrutinib (n = 1), tacrolimus (n = 1), TNF-inhibitor (n = 1)

### Neoehrlichia mikurensis PCR

All patients were diagnosed at the Department of Clinical Microbiology at the Sahlgrenska University Hospital, the National Reference Laboratory for neoehrlichiosis in Sweden, by using PCR. Total nucleic acid (NA) was extracted from 1 mL concentrated EDTA-plasma using the MagLEAD Extraction Robot (Precision Systemic Science, Matsudo, Chiba, Japan) combined with MagDEA Dx SV extraction kit. NA was eluted in 50 µL and analyzed by real-time PCR against a short segment of *groEL* as previously described [9].

### Mikurensis-ELISA (M-ELISA)

We performed an automated computational analysis to predict genes and search for protein homology in the *N. mikurensis* genome and identified seven candidate outer membrane proteins of the P44/Msp2. Two of these seven proteins exhibited higher intra-protein homology, while the others showed less overlap, the latter of which is advantageous in the context of developing a specific ELISA. We selected a small 19-kDa protein (comprising 174 amino acids) that appears to be specific to *N. mikurensis.* The P44/Msp2 protein sequence of the *N. mikurensis* SE24 clinical strain (GenBank accession no. CP066557.1; locus tag HL033_02330; nucleotide positions 577,689 to 578,213) [16], was used to produce synthetic peptide antigens (Peptides & Elephants GmbH, Henningsdorf, Germany): A long peptide (32–98) consisting of 67 amino acids (“long”) and a mixture of 41 overlapping peptides, each consisting of 15 amino acids (“mix”) covering the entire protein. This is based on the protein sequence available under GenBank accession number QXK93293.1.

Half-Area 96-well Polystyrene High Binding microplates (Corning Inc., New York, NY, USA) were coated with either 2 µg/mL solution of the mix peptides or 2 µg/mL of the long peptide in 0.05 M carbonate buffer, pH 9.6 and incubated overnight at 4 °C. All the following steps were performed at RT. The wells were washed twice with PBS containing 0.05% Tween 20 (PBS-T) (Sigma Aldrich, St. Louis, MO, USA) using a microplate washer (Agilent BioTek, Winooski, VT, USA) and blocked with 1% BSA (Sigma Aldrich) for 1 h. Serum and plasma samples were diluted 1:100 in 1% BSA and 50 µL was transferred to the coated plates in duplicates and incubated for 2 h. Next, the wells were washed thrice with PBS-T before the addition of goat anti-human IgM and IgG horseradish peroxidase (HRP)-conjugated secondary antibodies (Thermo Fisher Scientific, Bremen, Germany) diluted 0.5 µg/mL in 1% BSA for 2 h. Then, 50 µL of 3,3', 5,5' tetramethylbenzidine (TMB) substrate reagent set (Becton Dickinson, Franklin Lakes, NJ, USA) was added and incubated for 10 min. The reaction was stopped by adding 25 µL of 1 M H_2_SO_4_ and the optical density (OD) was measured at 450 nm using a SpectraMax Microplate Reader (Molecular Devices, San Jose, CA, USA).

### Borrelia serology

Commercial chemiluminescence immunoassays Liaison *Borrelia* IgM and IgG (DiaSorin, Saluggia, Italy) were used to measure antibodies to *Borrelia burgdorferi* sensu lato in serum.

### Statistics

The unpaired, two-tailed non-parametric Mann–Whitney test was used for groupwise comparisons and the Spearman rank test to calculate correlation coefficients. *P*-values < 0.05 were considered statistically significant. Prism software version 10.3.1 was employed (GraphPad Software, Boston, MA, USA).

## Results

### Blood donors with asymptomatic N. mikurensis infection

**Fig. 1:**
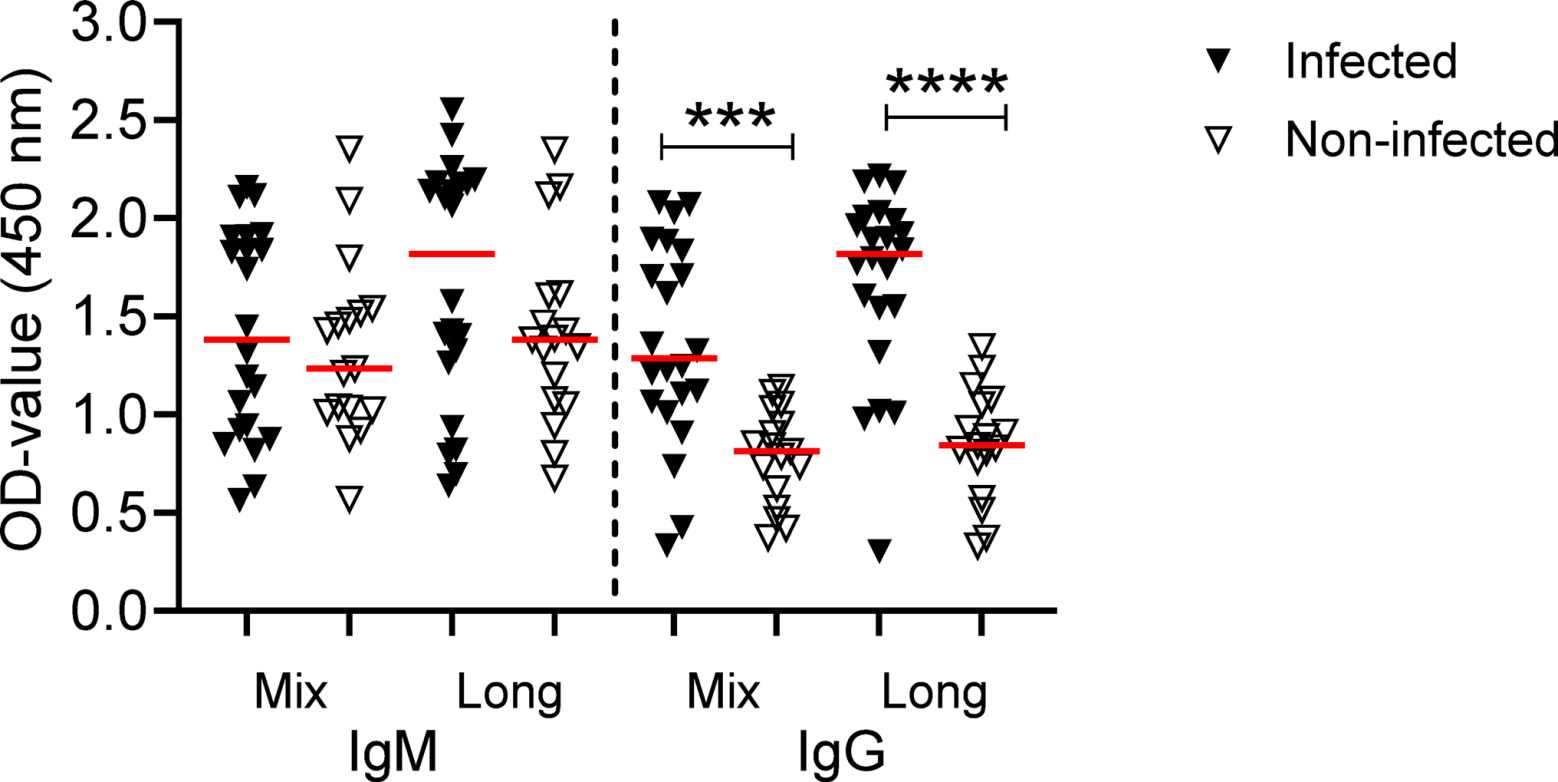
Asymptomatic blood donors infected with *N. mikurensis* have higher IgG antibody levels to *N. mikurensis* than non-infected blood donors. Plasma diluted 1:100 was analyzed for IgM and IgG antibody levels against the peptide mix and the long peptide antigen preparations of P44/Msp2 of *N. mikurensis*. The optical density (OD) values at absorbance 450 nm are shown. Black triangles show individual values for asymptomatically infected blood donors (*n* = 22) and white triangles show individual values for non-infected blood donors (*n* = 17). Red horizontal lines indicate medians. The Mann–Whitney test was used for statistical comparisons. ***P < 0.001; ****P < 0.0001

We first tested our new ELISA using plasma from blood donors who were asymptomatically infected with *N. mikurensis* and had tested positive by PCR and compared it with plasma from non-infected, PCR-negative blood donors. The blood donors were part of an ongoing study to determine whether blood transfusions can transmit *N. mikurensis* infection. None of the blood donors had overt symptoms of infection or disease. A total of 22 asymptomatically infected blood donors were identified and compared with non-infected blood donors (*n* = 17) for plasma antibody levels to *N. mikurensis*. The infected blood donors had significantly higher levels of IgG antibodies against the long and mix peptides of P44/Msp2 compared to the non-infected blood donors (Fig. 1). In contrast, no statistically significant differences were seen for the IgM antibody levels to either of the antigen preparations between the infected and non-infected blood donors (Fig. 1).

### Immunocompetent versus immunosuppressed neoehrlichiosis patients

**Fig. 2:**
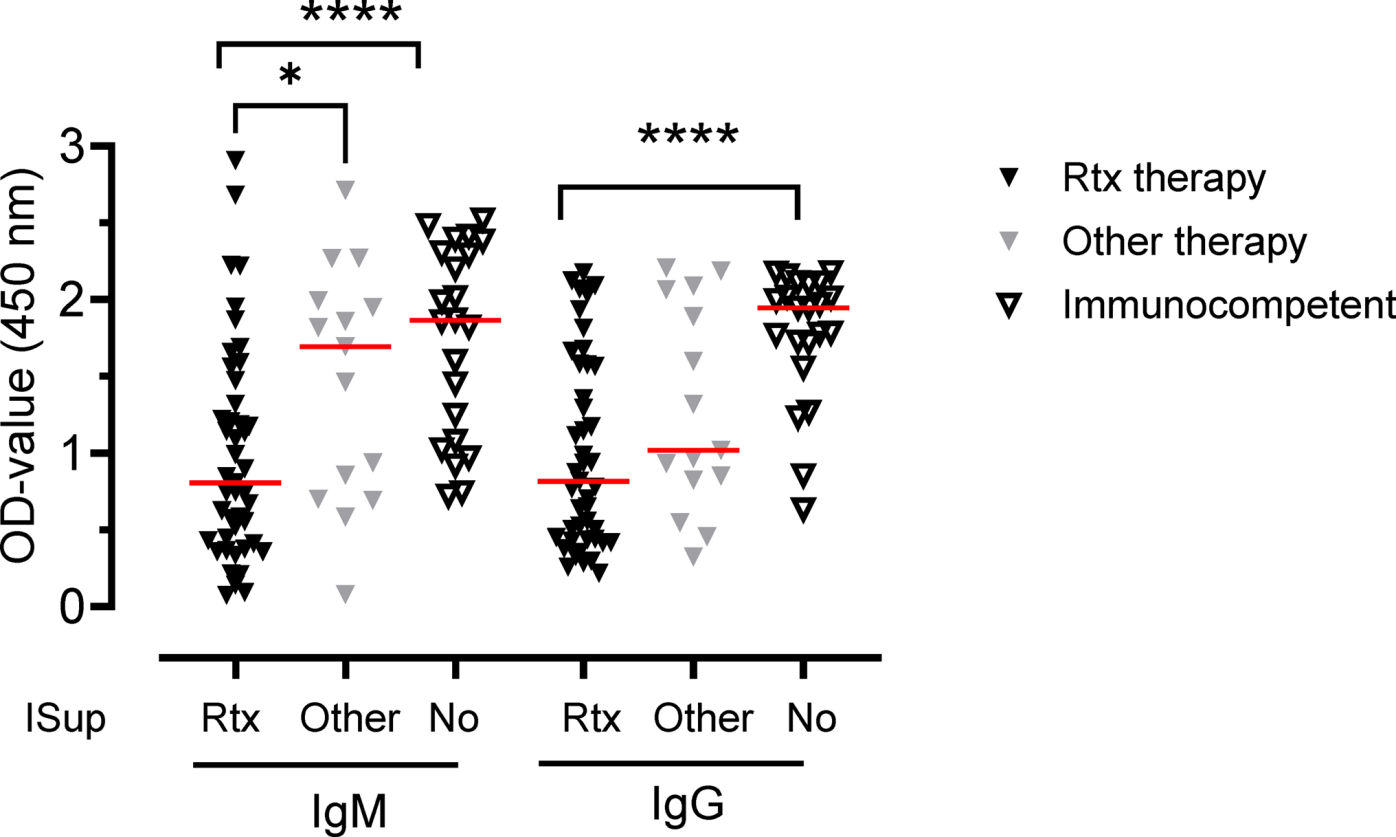
Immunosuppressed patients with neoehrlichiosis have lower IgG antibody levels than immunocompetent neoehrlichiosis patients. Neoehrlichiosis patients that were immunosuppressed (ISup) either due to rituximab therapy (Rtx, *n* = 45) or other therapy (Other, *n* = 15) were compared with immunocompetent patients with neoehrlichiosis (No, *n* = 22). Triangles show individual IgM and IgG levels to the long peptide of P44/Msp2 expressed as optical density (OD) values of sera diluted 1:100. Red horizontal lines indicate medians. The Mann–Whitney test was used for statistical comparisons. *P < 0.05; ****P < 0.0001

Eighty-two patients with symptomatic *N. mikurensis* infection, neoehrlichiosis, were classified as immunosuppressed (*n* = 60) or immunocompetent (*n* = 22), and analyzed for serum IgM and IgG antibody levels to the long peptide (Fig. 2). All the immunosuppressed patients had diseases requiring immunosuppressive therapy and were subdivided into two groups, one that had been treated with anti-CD20 therapy (Rtx, rituximab, *n* = 45) and one that had been treated with other immunosuppressants (Other, *n* = 15) (Table 1, Fig. 2). While both immunosuppressed groups had lower IgG antibody levels to *N. mikurensis* than the immunocompetent patients, it was only the rituximab group that had depressed IgM levels (Fig. 2). We also found no significant differences in IgM or IgG antibody levels to the long peptide between the immunocompetent patients with symptomatic *N. mikurensis* infection (Fig. 2) and the immunocompetent, asymptomatic blood donors infected with *N. mikurensis* (Fig. 1). The IgM responses to the long peptide were virtually identical: a median OD of 1.86 (range 1.07–2.33, 25th/75th percentile) for individuals in the symptomatic group and 1.82 (1.18/2.18) for the individuals in the asymptomatic group (NS). The corresponding IgG responses were 1.94 (range 1.68–2.10) versus 1.82 (range 1.49–1.99) for the symptomatic and asymptomatic groups (NS). The two groups of individuals had similar concentrations of *N. mikurensis* DNA in the blood, as assessed by real-time PCR Ct values (Table 1).

### Antibody titers to the long peptide

**Fig. 3:**
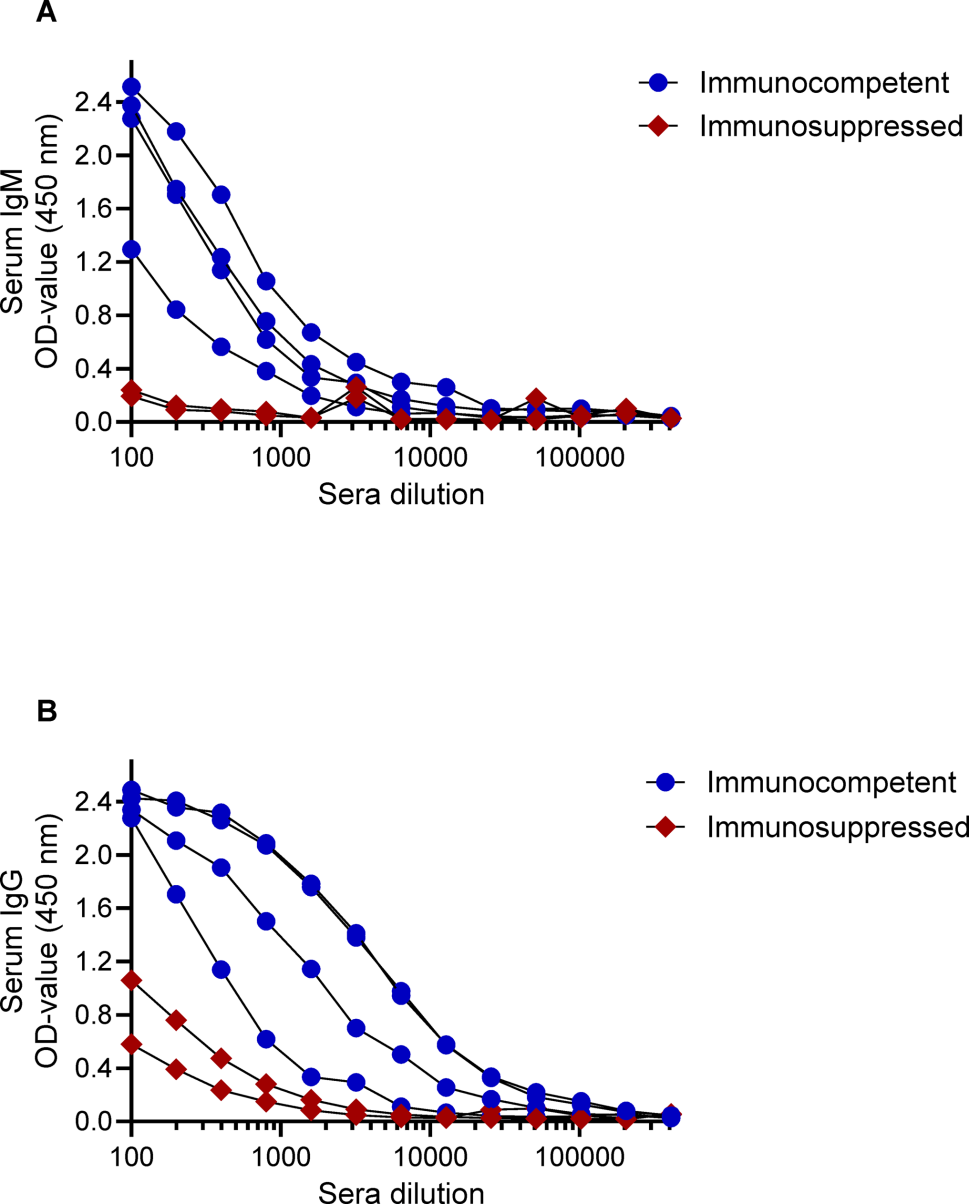
Immunocompetent individuals had significantly higher end-point serum antibody titers compared to immunosuppressed individuals. Two-fold serial dilutions of serum antibody levels to the long peptide antigen of P44/Msp2 of *N. mikurensis* are shown. The **A** panel indicates IgM responses, and the **B** panel indicates IgG responses. Optical density (OD) at 450 nm was measured. Blue symbols represent sera from immunocompetent individuals (*n* = 4), while red symbols represent immunosuppressed individuals (*n* = 2)

To assess the endpoint antibody titers to the long peptide of P44/Msp2, sera of four immunocompetent and two immunosuppressed individuals were titrated in two-fold serial dilutions (Fig. 3A, B). The endpoint IgM titer was 1:25,600 in the immunocompetent individuals and 1:1600 in the immunosuppressed individuals, representing a 16-fold difference. The IgG antibody endpoint titer was impressively high in the immunocompetent group: 1:204,800. In contrast, the endpoint IgG titer was only 1:3200 in the immunosuppressed group, corresponding to a 64-fold difference compared to the immunocompetent group.

### Non-infected children and cord blood samples

**Fig. 4:**
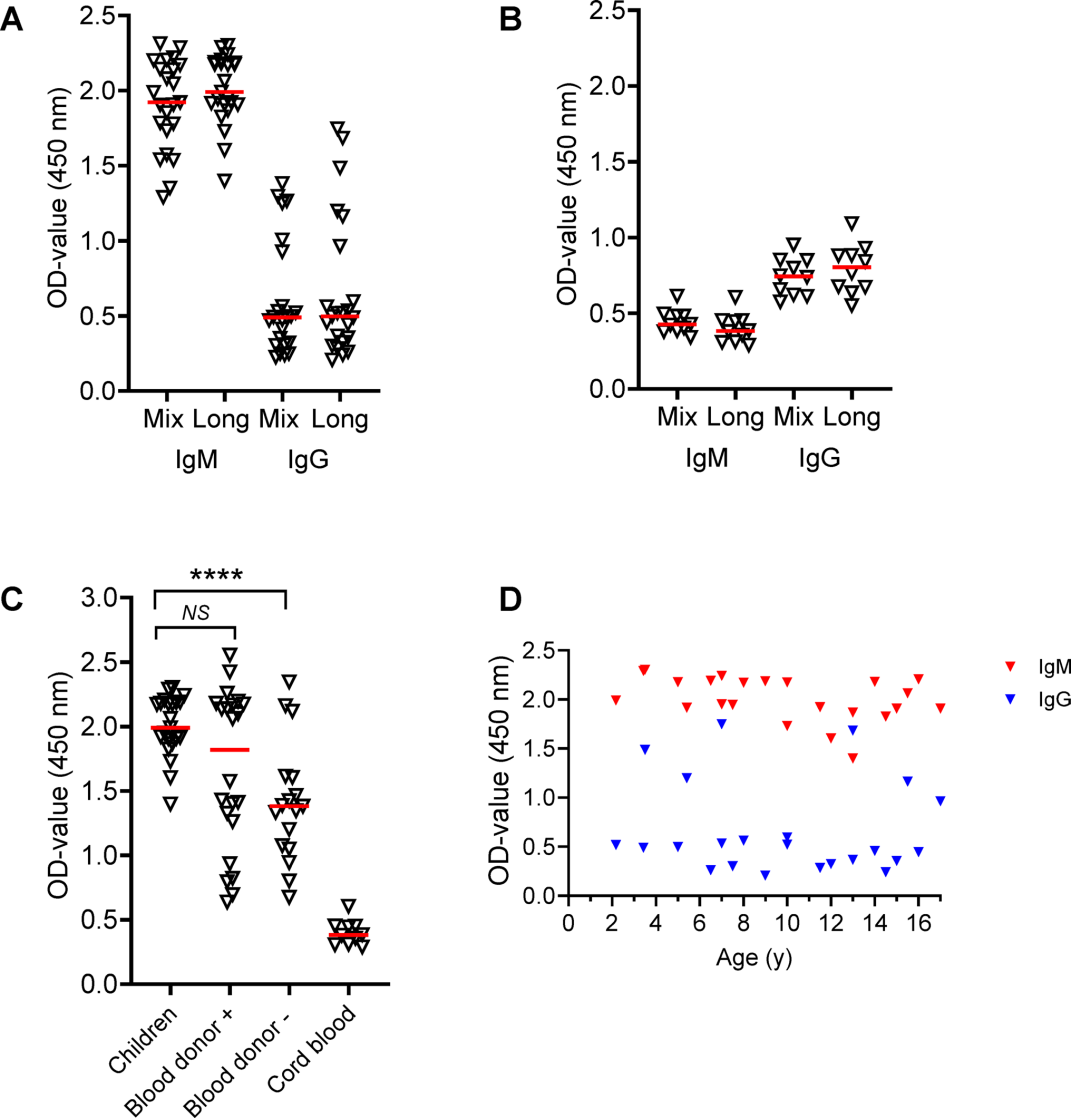
Non-infected children have high IgM reactivity to *N. mikurensis* in serum. **A** IgM and IgG antibody levels in the serum of non-infected children (*n* = 23) against the peptide mix and the long peptide antigen preparations of P44/Msp2 of *N. mikurensis*. **B** Antibody levels in cord blood samples (*n* = 10) to the antigens listed in “A”.** C** Comparison of the IgM antibody levels to the long peptide of P44/Msp2 in non-infected children (*n* = 23), infected blood donors (+ , *n* = 22), non-infected blood donors (-, *n* = 17) and cord blood samples (*n* = 10). Triangles show individual values and red lines indicate medians. The Mann–Whitney test was used for statistical comparisons. **D** Age-dependency of the IgM (red symbols) and IgG (blue symbols) antibody response to the long peptide of P44/Msp2 in the non-infected children (*n* = 23). ****P < 0.0001

To date, no child has been diagnosed with *N. mikurensis* infection. We tested biobanked blood sera that had been collected from 23 children aged 2–17 years immediately before minor surgery and whose serum samples had served as controls in separate studies of allergy and immune tolerance. All the sera tested negative for *N. mikurensis* by PCR. Remarkably, the children had very high levels of serum IgM to the P44/Msp2 antigen of *N. mikurensis* (Fig. 4A). This was suggestive of a natural IgM or polyreactive type of antibody response, a class of antibodies that are present in the newborn at birth and are generated independently of antigen stimulation. To address this issue, we tested cord blood samples (*n* = 10) but found very low levels of IgM to *N. mikurensis* (Fig. 4B). Remarkably, non-infected children had a marked IgM reactivity to P44/Msp2 of *N. mikurensis* in serum that was significantly higher than the IgM levels of non-infected adult blood donors and comparable to the IgM levels of infected blood donors (Fig. 4C). Lastly, we evaluated whether age influenced the antibody response of children to the *N. mikurensis* P44/Msp2 antigens but found that this was not the case for either the IgM or IgG response to *N. mikurensis* (Fig. 4D).

### Correlation of antibody reactivity to Borrelia burgdorferi s.l. and N. mikurensis

We evaluated if blood donors infected with *N. mikurensis* also had antibodies to *B. burgdorferi* s.l. and found that 7/16 (43%) of the *N. mikurensis*-infected blood donors had IgG antibodies, whereas none had IgM antibodies to *B. burgdorferi*. However, there were no correlations between the IgG antibody levels against *B. burgdorferi* and IgG antibodies against either the long peptide or the mix peptides of *N. mikurensis* P44/Msp2 (Spearman correlation coefficient *r* = 0.16, *P* = 0.54; *n* = 16 pairs). In contrast to the adults, none of the 23 control children with uniformly high IgM antibody levels to *N. mikurensis* had evidence of previous exposure to *B. burgdorferi*, as they all lacked both IgM and IgG antibodies to *B. burgdorferi* (data not shown).

## Discussion

Here we present the first ELISA for human IgM and IgG responses to *N. mikurensis*. In silico analysis of the annotated genomes of three clinical isolates of *N. mikurensis* guided our choice of bacterial antigen, a small outer membrane protein of the P44/Msp2 family with very low homology to other bacterial species. Unfortunately, it is technically very difficult to produce this type of protein in its entirety using recombinant technology, which is why we opted for peptides of different lengths. Although B-cell epitopes are generally considered to be discontinuous and conformational [21] in contrast to the short linear epitopes of T cells, we were fortunate in that our strategy of employing peptides worked well. Although both the pooled 15-amino acid peptide mixture and the long 67-amino acid peptide performed satisfactorily in the ELISA, the long peptide was the better antigen.

First, we analyzed the antibody responses of Swedish blood donors, asymptomatically infected with *N. mikurensis* and compared them with non-infected blood donors. The infected donors had significantly higher concentrations of IgG but not IgM antibodies to *N. mikurensis* than did the non-infected blood donors. It should be noted that although blood donors are not allowed to give blood if they are unwell, one blood donor infected with *N. mikurensis* experienced a marked improvement in health after the infection was eradicated. Similar testimonies of increased well-being among individuals with presumed asymptomatic carriage of *N. mikurensis* have been reported in both immunocompetent and immunosuppressed individuals [22, 23]. In this context, it may seem counterintuitive that the levels of both IgM and IgG to *N. mikurensis* did not differ between the immunocompetent patients with symptomatic disease and the asymptomatic infected blood donors. However, this finding is consistent with the fact that these two groups of individuals had identical bacterial loads in the blood, as reflected by the Ct values of the *N. mikurensis* PCR (Table 1).

As expected, immunosuppressed patients with symptomatic *N. mikurensis* infection, neoehrlichiosis, had lower antibody concentrations to *N. mikurensis* than the immunocompetent patients with neoehrlichiosis. This was evident for patients treated with rituximab, who had lower levels of both IgM and IgG antibodies compared with the immunocompetent patients. Patients treated with other types of immunosuppressive agents directed against T cells, cytokines or B-cell targets other than CD20, had lower levels of IgG, but not IgM, compared with the immunocompetent group of patients. In particular, rituximab therapy leads to depletion of the naïve and unswitched B cell populations that normally develop into IgM-secreting B cells [24].

It is not understood why there are no reports of children being infected with *N. mikurensis.* Children and middle-aged people are the main demographic groups afflicted by *Borrelia* infections [14], presumably because they are more active outdoors and more exposed to ticks than younger adults. Our previous hypothesis was that children may be protected from *N. mikurensis* infection by virtue of their smooth, non-arteriosclerotic blood vessel walls [7]. Here, we propose a new theory based on our finding of strikingly elevated levels of IgM antibodies in the blood of children who appear to have had little contact with ticks, given their complete lack of antibodies against *Borrelia*. We can only speculate about the origin of these antibodies—they were not present in the neonates´ cord blood and therefore may not constitute a major class of steady-state natural IgM antibodies, but we cannot rule out the possibility that the most immature B-cell populations present in the cord blood may be intrinsically primed to produce this class of natural or polyreactive antibodies in response to certain stimuli. Natural IgM antibodies are synthesized in an antigen-independent fashion and provide broad anti-microbial defense [25]. We have previously shown that natural IgM antibodies do not appear to have a prominent role in the immune defense against *N. mikurensis* in adults [26]. The source of the IgM antibodies to *N. mikurensis* detected in children may be polyreactive antibodies generated after exposure to other microbial antigens or even self-antigens. Polyreactive IgM antibodies have been shown to confer protection against murine ehrlichiosis, an infection caused by *Ehrlichia muris*, which belongs to the same family of *Anaplasmataceae* as *N. mikurensis*. An intriguing feature of these polyreactive protective murine IgM antibodies is that they recognize a 19 kDa protein of *E. muris*, and that the same type of IgM antibodies are also found in human ehrlichiosis [27]. The fact that only a few children (5/20) showed elevated IgG levels could indicate previous exposure to *N. mikurensis,* cross-reactive responses to *A. phagocytophilum,* or exposure to an entirely different antigen.

A major limitation of our study is the lack of negative controls, which makes it impossible to provide the specificity, sensitivity and cut-off levels of the ELISA. We had expected that children might constitute negative controls since neoehrlichiosis has never been reported in children. Our analyses revealed that our cohort of children had strikingly elevated levels of serum IgM, which could explain why children appear immune to this new pathogen. It is impossible to find persons in Sweden who have not been bitten by ticks and *N. mikurensis* is carried by up to 15% of the ticks in Sweden [6]. *N. mikurensis* is also widely spread in Europe and northern Asia [15]. Moreover, we cannot rule out serological cross-reactivity with *A. phagocytophilum* or previous exposure to *A. phagocytophilum* as a confounding factor [19], which would exclude using samples from persons residing in North America as negative controls. This limitation can only properly be addressed by studies of very rare serum samples from permanent residents of tick-free regions such as the Arctic and possibly the southern hemisphere.

Prior to the development of this ELISA, the only published method for studying antibody responses to *N. mikurensis*, was our own image flow cytometry-based method, in which experimentally infected tick or endothelial cell lines were probed with immune sera [6]. The advantage of the latter method is that it allows the measurement of antibodies to the whole bacterium, but at the cost of being extremely cumbersome and only allowing the analysis of 8–10 samples per day. The ELISA allows high-throughput analysis of antibody responses to *N. mikurensis*. Hopefully, the ELISA will help to bridge the gaps of knowledge regarding human infections with *N. mikurensis,* such as the seroprevalences of this infection in different populations, the longevity of the antibody response, and whether protective levels of antibodies can be identified. It should be emphasized that the ELISA is, in its current state, not suitable for diagnostic purposes.

## Data Availability

No datasets were generated or analysed during the current study.

## References

[1] Fehr JS, Bloemberg GV, Ritter C, Hombach M, Luscher TF, Weber R, Keller PM (2010) Septicemia caused by tick-borne bacterial pathogen Candidatus Neoehrlichia mikurensis. Emerg Infect Dis 16:1127–1129. 10.3201/eid1607.091907 20587186 10.3201/eid1607.091907 PMC3358111

[2] Welinder-Olsson C, Kjellin E, Vaht K, Jacobsson S, WenneråS C (2010) First case of human “Candidatus Neoehrlichia mikurensis” infection in a febrile patient with chronic lymphocytic leukemia. J Clin Microbiol 48:1956–1959. 10.1128/jcm.02423-09 20220155 10.1128/JCM.02423-09 PMC2863919

[3] Von Loewenich FD, GeißdöRfer W, Disqué C, Matten J, Schett G, Sakka SG, Bogdan C (2010) Detection of “Candidatus Neoehrlichia mikurensis” in two patients with severe febrile illnesses: evidence for a European sequence variant. J Clin Microbiol 48:2630–2635. 10.1128/jcm.00588-10 20519481 10.1128/JCM.00588-10 PMC2897504

[4] Grankvist A, Andersson PO, Mattsson M, Sender M, Vaht K, Hoper L, Sakiniene E, Trysberg E, Stenson M, Fehr J, Pekova S, Bogdan C, Bloemberg G, Wenneras C (2014) Infections with the tick-borne bacterium “Candidatus Neoehrlichia mikurensis” mimic noninfectious conditions in patients with B cell malignancies or autoimmune diseases. Clin Infect Dis 58:1716–1722. 10.1093/cid/ciu189 24647019 10.1093/cid/ciu189

[5] Höper L, Skoog E, Stenson M, Grankvist A, Wass L, Olsen B, Nilsson K, Mårtensson A, Söderlind J, Sakinis A, Wennerås C (2021) Vasculitis due to Candidatus Neoehrlichia mikurensis: a cohort study of 40 Swedish patients. Clin Infect Dis 73:e2372–e2378. 10.1093/cid/ciaa1217 32818961 10.1093/cid/ciaa1217

[6] Wass L, Grankvist A, Bell-Sakyi L, Bergström M, Ulfhammer E, Lingblom C, Wennerås C (2019) Cultivation of the causative agent of human neoehrlichiosis from clinical isolates identifies vascular endothelium as a target of infection. Emerg Microbes Infect 8:413–425. 10.1080/22221751.2019.1584017 30898074 10.1080/22221751.2019.1584017 PMC6455172

[7] Wennerås C, Wass L, Bergström B, Grankvist A, Lingblom C (2024) Ten years of detecting Neoehrlichia mikurensis infections in Sweden: demographic, clinical and inflammatory parameters. Eur J Clin Microbiol Infect Dis 43:2083–2092. 10.1007/s10096-024-04909-5 39136831 10.1007/s10096-024-04909-5 PMC11535080

[8] Boyer PH, Baldinger L, Degeilh B, Wirth X, Kamdem CM, Hansmann Y, Zilliox L, Boulanger N, Jaulhac B (2021) The emerging tick-borne pathogen Neoehrlichia mikurensis: first French case series and vector epidemiology. Emerg Microbes Infect 10:1731–1738. 10.1080/22221751.2021.1973347 34432610 10.1080/22221751.2021.1973347 PMC8425734

[9] Grankvist A, Sandelin LL, Andersson J, Fryland L, Wilhelmsson P, Lindgren P-E, Forsberg P, Wennerås C (2015) Infections with Candidatus Neoehrlichia mikurensis and cytokine responses in 2 persons bitten by ticks, Sweden. Emerg Infect Dis 21:1462–1465. 10.3201/eid2108.150060 26197035 10.3201/eid2108.150060 PMC4517700

[10] Labbé Sandelin L, Olofsson J, Tolf C, Rohlén L, Brudin L, Tjernberg I, Lindgren P-E, Olsen B, Waldenström J (2022) Detection of *Neoehrlichia mikurensis* DNA in blood donors in southeastern Sweden. Infect Dis Lond. 10.1080/23744235.2022.2087732 35724266 10.1080/23744235.2022.2087732

[11] Markowicz M, Schötta A-M, Höss D, Kundi M, Schray C, Stockinger H, Stanek G (2021) Infections with tickborne pathogens after tick bite, Austria, 2015–2018. Emerg Infect Dis. 10.3201/eid2704.203366 33755546 10.3201/eid2704.203366 PMC8007293

[12] Welc-Falęciak R, Siński E, Kowalec M, Zajkowska J, Pancewicz SA (2014) Asymptomatic “Candidatus Neoehrlichia mikurensis” infections in immunocompetent humans. J Clin Microbiol 52:3072–3074. 10.1128/jcm.00741-14 24899023 10.1128/JCM.00741-14 PMC4136151

[13] Dahlberg AO, Aase A, Reiso H, Midgard R, Quarsten H (2025) Detection of *Neoehrlichia mikurensis* in 11 persons who attribute their persistent health complaints to a tick-borne disease. Ticks Tick-borne Dis 16:102391. 10.1016/j.ttbdis.2024.102391 39265459 10.1016/j.ttbdis.2024.102391

[14] Goren A, Mysterud A, Jore S, Viljugrein H, Bakka H, Vindenes Y (2023) Demographic patterns in Lyme borreliosis seasonality over 25 years. Zoonoses Public Health 70:647–655. 10.1111/zph.13073 37458418 10.1111/zph.13073

[15] Wennerås C (2015) Infections with the tick-borne bacterium Candidatus Neoehrlichia mikurensis. Clin Microbiol Infect 21:621–630. 10.1016/j.cmi.2015.02.030 25770773 10.1016/j.cmi.2015.02.030

[16] Grankvist A, Jaén-Luchoro D, Wass L, Sikora P, Wennerås C (2021) Comparative genomics of clinical isolates of the emerging tick-borne pathogen *Neoehrlichia mikurensis*. Microorganisms 9:1488. 10.3390/microorganisms9071488 34361922 10.3390/microorganisms9071488 PMC8303192

[17] Zhi N, Rikihisa Y, Kim HY, Wormser GP, Horowitz HW (1997) Comparison of major antigenic proteins of six strains of the human granulocytic ehrlichiosis agent by Western immunoblot analysis. J Clin Microbiol 35:2606–2611. 10.1128/jcm.35.10.2606-2611.1997 9316916 10.1128/jcm.35.10.2606-2611.1997 PMC230019

[18] Grankvist A, Moore ER, Svensson Stadler L, Pekova S, Bogdan C, Geissdorfer W, Grip-Linden J, Brandstrom K, Marsal J, Andreasson K, Lewerin C, Welinder-Olsson C, Wenneras C (2015) Multilocus sequence analysis of clinical “Candidatus Neoehrlichia mikurensis” strains from Europe. J Clin Microbiol 53:3126–3132. 10.1128/JCM.00880-15 26157152 10.1128/JCM.00880-15 PMC4572549

[19] Wass L, Grankvist A, Mattsson M, Gustafsson H, Krogfelt K, Olsen B, Nilsson K, Martensson A, Quarsten H, Henningsson AJ, Wenneras C (2018) Serological reactivity to *Anaplasma phagocytophilum* in neoehrlichiosis patients. Eur J Clin Microbiol Infect Dis 37:1673–1678. 10.1007/s10096-018-3298-3 29948363 10.1007/s10096-018-3298-3 PMC6133046

[20] Wenneras C, Aranburu A, Wass L, Grankvist A, Staffas A, Soboli A, Martensson IL, Fogelstrand L, Lewerin C (2023) Infection with *Neoehrlichia mikurensis* promotes the development of malignant B-cell lymphomas. Br J Haematol 201:480–488. 10.1111/bjh.18652 36650117 10.1111/bjh.18652

[21] Sivalingam GN, Shepherd AJ (2012) An analysis of B-cell epitope discontinuity. Mol Immunol 51:304–309. 10.1016/j.molimm.2012.03.030 22520973 10.1016/j.molimm.2012.03.030 PMC3657695

[22] Quarsten H, Grankvist A, Høyvoll L, Myre IB, Skarpaas T, Kjelland V, Wenneras C, Noraas S (2017) CandidatusNeoehrlichia mikurensis and *Borrelia burgdorferi* sensu lato detected in the blood of Norwegian patients with erythema migrans. Ticks Tick-borne Dis 8:715–720. 10.1016/j.ttbdis.2017.05.004 28539197 10.1016/j.ttbdis.2017.05.004

[23] Quarsten H, Salte T, Lorentzen ÅR, Hansen IJW, Hamre R, Forselv KJN, Øines Ø, Wennerås C, Noraas S (2021) Tick-borne pathogens detected in the blood of immunosuppressed Norwegian patients living in a tick-endemic area. Clin Infect Dis 73:e2364–e2371. 10.1093/cid/ciaa971 32662513 10.1093/cid/ciaa971

[24] Kridin K, Ahmed AR (2020) Post-rituximab immunoglobulin M (IgM) hypogammaglobulinemia. Autoimmun Rev 19:102466. 10.1016/j.autrev.2020.102466 31917267 10.1016/j.autrev.2020.102466

[25] Grönwall C, Vas J, Silverman GJ (2012) Protective roles of natural IgM antibodies. Front Immunol. 10.3389/fimmu.2012.00066 22566947 10.3389/fimmu.2012.00066 PMC3341951

[26] Wennerås C, Goldblatt D, Zancolli M, Mattsson M, Wass L, Hörkkö S, Rosén A (2017) Natural IgM antibodies in the immune defence against neoehrlichiosis. Infect Dis 49:809–816. 10.1080/23744235.2017.1347815 10.1080/23744235.2017.1347815 28682152

[27] Jones DD, DeIulio GA, Winslow GM (2012) Antigen-driven induction of polyreactive IgM during intracellular bacterial infection. J Immunol 189:1440–1447. 10.4049/jimmunol.1200878 22730531 10.4049/jimmunol.1200878 PMC3401281

